# Single shot femtosecond laser nano-ablation of CVD monolayer graphene

**DOI:** 10.1038/s41598-018-32957-3

**Published:** 2018-10-02

**Authors:** A. Gil-Villalba, R. Meyer, R. Giust, L. Rapp, C. Billet, F. Courvoisier

**Affiliations:** FEMTO-ST institute, Univ. Bourgogne Franche-Comté, CNRS, 15B avenue des Montboucons, 25030 Besançon Cedex, France

## Abstract

We investigate ablation of CVD monolayer graphene by femtosecond pulses in the single shot regime. We show that the ablation probability of flat graphene drastically reduces for small illumination diameters even if the ablation threshold is exceeded. However, the presence of graphene wrinkles enhances the ablation probability. This is interpreted in terms of electron and energy diffusion within the graphene layer. This differentiated behavior is a drawback for single shot laser nanopatterning. The morphology of the holes with minimal diameter depends on the fluence distribution at ablation threshold. Strong fluence gradients due to strong focussing produce an explosive folding of graphene during ablation.

## Introduction

Since its isolation, graphene has attracted attention due to its properties as a material for next generation technologies. Graphene has potential applications in different fields such as optoelectronics, photonics or photovoltaics^1^. Chemical vapour deposition (CVD) has emerged as a promising technology to produce graphene for large-scale electronic devices because it can be combined with standard wafer-scale lithographic methods; it is compatible with integrated circuit fabrication processes with low cost and high efficiency. Most graphene-based devices for photonic and optoelectronic applications will require nanopatterning arrays of holes with diameters ranging from 100 nm to 1 µm^2,3^.

Different methods of patterning graphene at nanometer scale have been developed including focused ion beam^4^, electron beam lithography with reactive ion etching^5,6^, UV nanoimprint lithography^7^, and direct etching with an electron beam in a transmission electron microscope^8^. Most of these procedures require vacuum and multiple steps, which are difficult to use for mass production of graphene-based devices.

Ultrafast laser processing is a promising technique because it requires no vacuum, it is single step, easily reconfigurable and large areas can be rapidly patterned. In addition, ultrafast pulses generate small heat affected zones in the surrounding materials. Importantly, the non-ablated regions of graphene maintain the pristine structure, which was confirmed by Raman microscopy^9–11^.

The marked threshold effect of the femtosecond laser ablation process enables sub-spot and even sub-wavelength processing. For instance, hole diameters down to 100 nm have been processed at the surface of transparent materials or in thin films (polymers, metallic films etc) using ultrashort laser pulses at 800 nm wavelength^12,13^. In this context, direct laser ablation of graphene has been investigated in the single shot regime for holes on the order of 1 to 10 µm^14^, and in the multishot regime for smaller diameters down to below 500 nm^10,15,16^. Nanoribbons with width in the sub 100-nm range have been achieved in thermal accumulation regime with femtosecond pulses^17^. However, the processing speed was incompatible with industrial scale fabrication.

Here, our objective is to characterize ablation characteristics in the single shot regime close to ablation threshold with tight and loose focussing conditions. The single shot regime is a priori more interesting in terms of processing speed. However, in this regime, a recent study of the ablation probability with multiple spots determined a strong decrease of the ablation probability when the beam diameter was decreasing below ~1 µm^18^.

**Figure 1:**
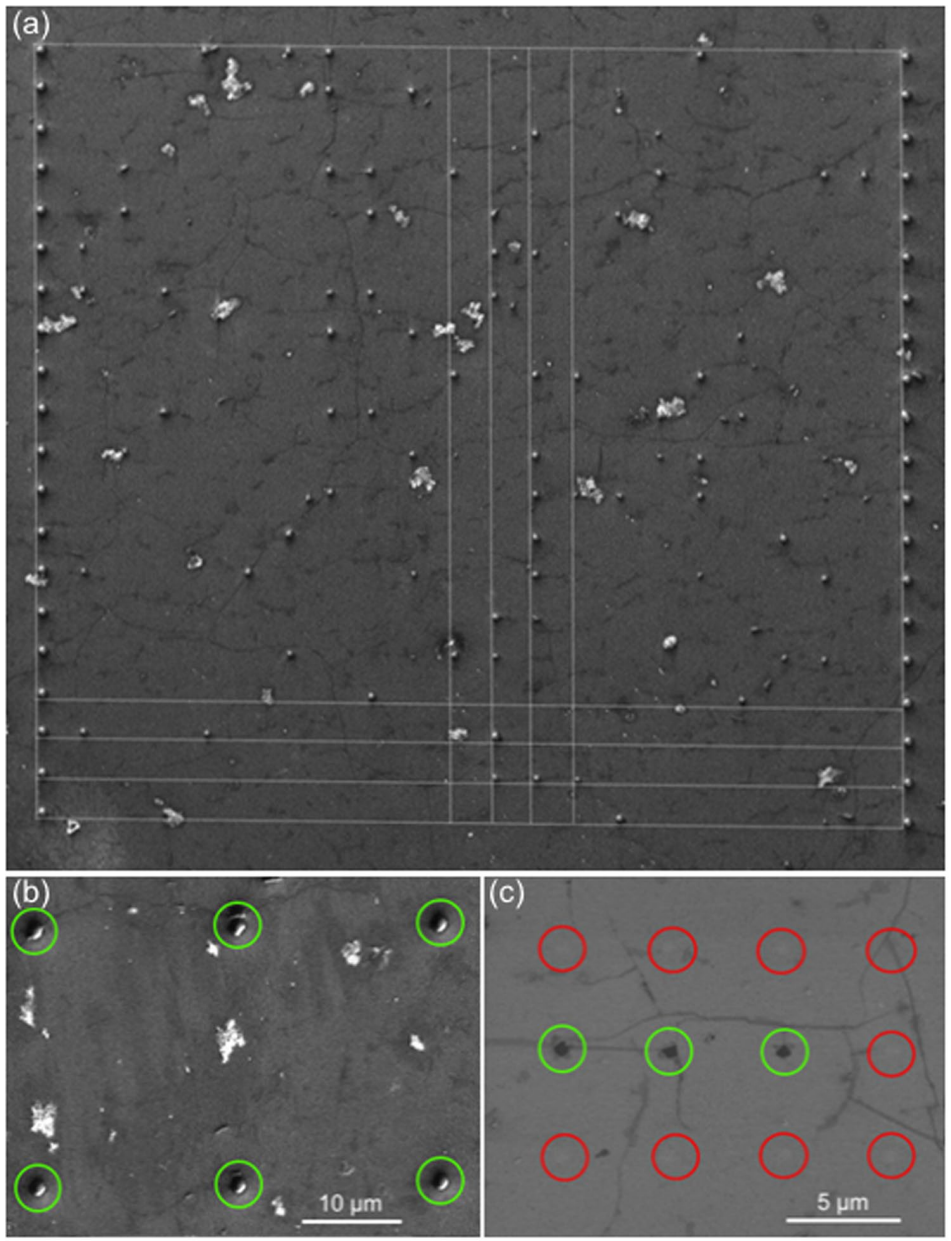
(**a**) SEM image of matrix of equidistant laser illuminated spots with zero-th order Bessel beam with peak fluence of (**b**) 0.72 J/cm^2^ and (**c**) 0.36 J/cm^2^. Red circles indicate illuminated but non-ablated sites and green circles indicates illuminated and ablated sites. Correlation between ablated holes and presence of wrinkles (dark grey lines on SEM image) is obvious for the lower pulse energy.

**Figure 2:**
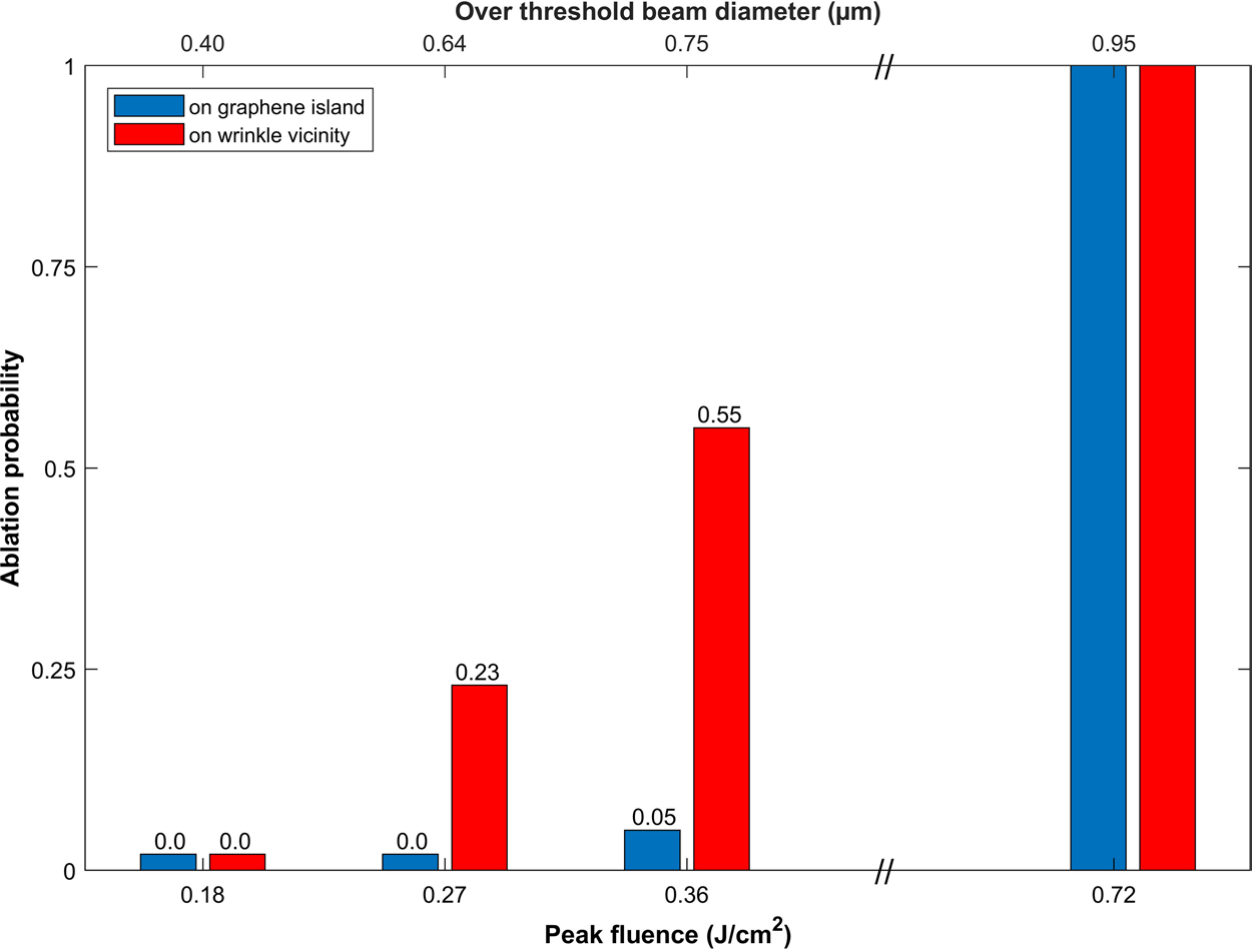
Ablation probability comparison between illuminated site in a graphene island (red) and in the vicinity of a wrinkle (blue). The Bessel beam cone angle is 26°.

The presence of defects is important for laser processing, which is particularly the case for CVD graphene. We will investigate the influence on ablation statistics of the presence of defects in form of graphene wrinkles in the graphene layer. Large scale fabrication of graphene by CVD has inherent formation of wrinkles during transfer, which are nanometric foldings of the graphene layer which impacts on its local transport properties^19,20^ and therefore on electrical and optical properties. Graphene wrinkles can be easily visualized under Scanning Electron Microscopy (SEM) (see ref.^19^ Fig. 1 and^20^ Fig. 2). We will show that while they have no noticeable influence on the morphology of the ablated site, graphene wrinkles strongly enhance the ablation probability at small beam diameters in comparison with what will be called here “flat graphene islands”, where graphene does not show wrinkles observable under SEM imaging.

We show that ablation probability on flat island vanishes as soon as the fluence distribution is sufficiently small. The high conductivity and diffusion coefficients of graphene are probably the cause for this behaviour. Then, we will investigate the hole diameter as a function of input fluence distribution for tight and loose focussing. Finally, we will compare the morphologies of the holes produced: no influence of the presence of wrinkles was noticeable. In contrast, the fluence distribution strongly affects the crater morphology: we observe graphene folding in the strong focussing conditions.

## Methodology

A technical difficulty to investigate near-threshold ablation, when the targeted crater diameters are in the ~100 nm range is to precisely maintain the focus of the beam on the sample^12,13^. Here, we overcome this difficulty by using nondiffracting Bessel beams^21^. These beams possess a narrow focus which extends longitudinally over several tens of micrometers, well above the Rayleigh range of a Gaussian beam with equivalent waist^22,23^. We have compared ablation with two different spot sizes: 0.65 µm FWHM (tight focussing, Bessel cone angle of 26°) and 1.6 µm (looser focussing, Bessel cone angle of 10°).

We also need a quantitative element to compare between illumination conditions. Since the fluence distribution is different between the beams, we defined the *over-threshold beam diameter* as the lateral diameter of the central Bessel beam core over which the local fluence exceeds the ablation threshold, which was previously determined at 139 mJ/cm^2^ for 130 fs pulses^18^. This was measured in conditions where the hole diameter exceeds 1 µm and is consistent with literature^9–11,15,16^. For large diameters, the effect of fluence distribution within the over-threshold spot has a negligible effect, so that different focussing conditions lead to the same damage size if the over-threshold diameter is the same. We will investigate here conditions where the over-threshold diameter is on the order of 1 µm and below.

## Setup

In our experiments, we used an amplified Ti:Sapphire laser system delivering 130 fs pulses at 800 nm. The laser operates at 5 kHz repetition rate and pulse-picking was implemented to operate in single-shot regime. Bessel beams were generated by applying a phase mask onto a Spatial Light Modulator (SLM, Hamamatsu PAL-SLM) to generate a virtual axicon. The SLM is associated to an optical demagnification system (1/278 factor). The setup includes spatial Fourier filtering and was described in detail in ref.^24^. We used two Bessel beams with different cone angle, i.e. the angle made by the geometrical rays with the optical axis: 26° and 10°. The Bessel beam with 26° cone angle has a diameter of 0.65 µm FWHM and its Bessel zone extends over ~27 µm. The Bessel beam with 10° cone angle has a diameter of 1.6 µm FWHM (ie nearly 2.5 times larger than the previous one), extending over ~75 µm. The graphene sample is CVD-grown graphene monolayer on Corning Eagle-XG AMLCD glass substrate. We stress that the fluences used (<0.72 J/cm^2^) remained always below the damage threshold of the substrate. (After reproducing the same series on the native substrate, no damages were observed under optical microscopy and SEM.) The graphene monolayer was facing the incident beam, placed at the peak of the longitudinal intensity distribution within 2 µm precision.

To investigate the ablation statistics, we repeated the illumination 400 times in a matrix, where the sample was moved between each illumination. The illumination sites were determined on SEM images from the positions of holes performed at fluence well above threshold on two lines, on the left and right sides of the matrix as shown in Fig. 1(a). These holes used as marks have a diameter of ~1 µm. The illumination sites were spaced by 5 µm one of each other so that they are uncorrelated. Indeed, the intensity drops from the center of the beam to 5 µm away down to 6%, so that the intensity due to the Nth shot on the previous (N-1) site is only about 10% of the ablation threshold. Previous experiments did not show any correlation even at shorter distance^18^ and no statistical difference has been observed between the first and last point of the series.

## Results

Figure 1(b,c) shows high resolution SEM images in the matrices obtained for peak fluences of 0.7 J/cm^2^ (Fig. 1b) and 0.4 J/cm^2^ (Fig. 1c) with 26° cone angle. In the first case, the ablation probability is 100%, while in the second case, it is immediately apparent that no ablation occurred on flat graphene islands, but did occur more often when the illumination is in the vicinity of wrinkles.

We have therefore discriminated the two different kinds of sites during the analysis of the SEM images for a 26° cone angle. The criterion for the vicinity of the wrinkle was chosen to be a disk of 0.5 µm radius around the centre of the laser illumination site. Yet the value of 0.5 µm is arbitrary, we noticed that this was a threshold marking the difference between flat graphene islands and wrinkled areas. Figure 2 summarizes the results, where the ablation probability is plotted as a function of input peak fluence, which we also expressed as the over-threshold diameter. The latter scale is shown on the top of the figure.

From 0.95 µm of over-threshold beam diameter and above, the ablation is 100%. The probability rapidly drops for diameters below this value. Importantly, this value is the same as was determined with lower focussing conditions (9° interference, larger spots FWHM in ref.^18^). But the comparison between the sites reveals that the ablation probability very quickly drops to zero for flat graphene islands, while wrinkles enhance the ablation probability so that it still exceeds 20% at over-threshold beam diameter of 0.6 µm.

**Figure 3:**
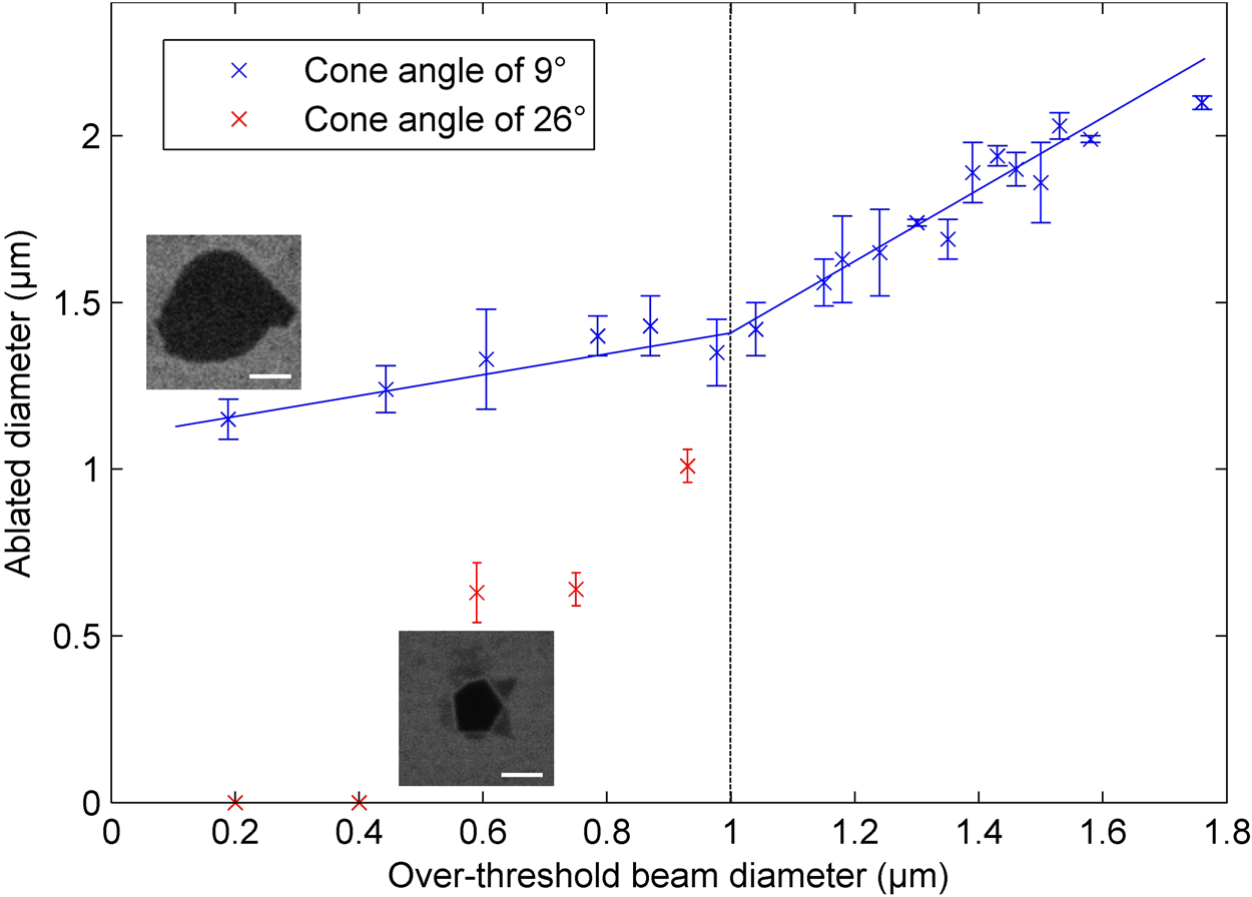
Diameter of the holes as a function of over-threshold laser beam diameter for two different Bessel beams with cone angles of 9° and 26° (see text). The insets correspond to the minimal ablated hole for each cone angle. White bars correspond to 0.5 µm.

We further investigate the evolution of the diameter of the holes with pulse energy or equivalently, as a function of *over-threshold diameter*, as shown in Fig. 3. The average diameter of the holes was determined as twice the square root of the hole surface divided by π. For a Bessel beam with 26° cone angle, the minimal diameter achievable was ~650 nm for over-threshold beam diameters between 600 and 800 nm. No ablation was observed for over-threshold diameters below 600 nm. We have repeated the experiment with a beam with 9° cone angle as shown with blue markers in the same figure. Above an over-threshold diameter of 1 µm, a linear dependence with slope 1 is observed between the ablated diameter and the over-threshold diameter, as can be expected for the threshold-like behaviour of ultrafast laser ablation. In contrast, below over-threshold diameter of ~1 µm, the diameter of the holes saturates slightly above 1 µm. No ablation could be observed below 1 µm in diameter.

**Figure 4:**
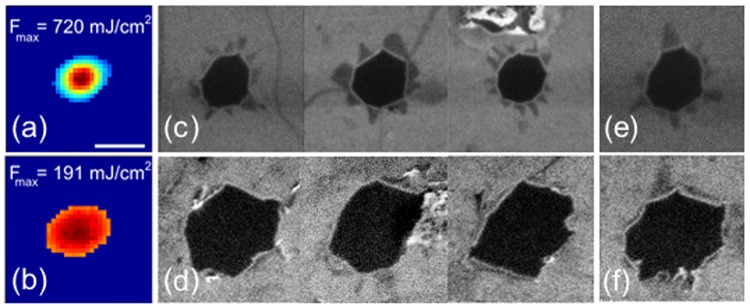
Experimentally characterized over-threshold fluence beam (**a**,**b**) and SEM images of ablated holes for a pulse energy of 20.0 nJ for cone angle of (top) 26° and (bottom) 9°. This corresponds to the same value of over threshold diameter, which is ~1 µm here. White scale bar correspond to 1 µm. All images are at the same scale. (**c**,**d**) SEM images of holes occuring in the vicinity of a wrinkle. (**e**,**f**) SEM images of holes occuring on graphene islands.

The morphology of the holes showed no dependence on the presence of wrinkle, but strongly depends on the focusing conditions. Figure 4 compares SEM images of ablated holes: the first line corresponds to a cone angle of 26° while the second is for 9° cone angle. The over-threshold beam diameter is quasi-identical for the two conditions and is ~1 µm. In (c,d) ablation occurred in the vicinity of a wrinkle while in (e,f) ablation occurred on a graphene island. It is apparent that the presence of wrinkles shows no significant effect on the morphology, but strong presence of graphene folding is observed for the strong focussing conditions. In this case, we note that the total energy enclosed within the above-threshold disk is twice higher than with 9° illumination (0.46 nJ enclosed against 0.2 nJ). This difference along with the existence of a strong fluence gradient might explain the difference in the folding behaviour.

## Discussion

In the ablation process, what determines the amplitude of the phase transformation is the deposited energy *density* at the time of the phase transformation. Our experiments can be understood in terms of diffusion of the laser deposited energy. For large values of the over-threshold beam diameter, energy diffusion has a minor effect on the distribution since spatial gradients are small. In this case, a fluence threshold criterion is enough to describe the area that will be ablated. In contrast, below a value of ~1 µm (which is on the same order of magnitude as the mean-free path of free-electrons in graphene^1^), high gradients are involved, so that free-electron diffusion and/or heat diffusion are non-negligible. This explains the deviations observed in Fig. 3 for over-threshold diameters below 1 µm: the ablated area does not correspond anymore to the surface delimited by the threshold fluence (slope of 1). The key benefits of graphene in terms of high electronic and thermal conductivity coefficients become, in the case of ablation, drawbacks for energy confinement and for the efficiency of the single shot ablation process. In contrast, processing using multiple illuminations is expected to yield better results in terms of resolution and ablation probability because the defects produced in the lattice by previous shots can increase the confinement for further pulses^10,11,13^.

The difference of ablation statistics if the illumination occurs close to a wrinkle or on a flat graphene island can be understood in this framework. Indeed, energy diffusion outside the “over-threshold” area is reduced in the vicinity of a graphene wrinkle because it acts as a barrier of potential for conduction band electrons. Phonon density is also reduced at defects sites. Energy confinement is therefore enhanced by the wrinkle, which explains why ablation is possible with lower amounts of deposited energy than for the case of flat graphene islands. We also note that our study could not discriminate point defects or grain boundaries which are at scales well below what can be observed under SEM. These defects will also produce similar energy confinement and increase of the ablation probability.

We finally note that for the smallest holes obtained at 26° cone angle, the surface of the folded structure exactly corresponds to the ablated one: negligible material amount was actually removed from the sample in these conditions, since the explosion made the graphene layer to fold. The extreme mechanical toughness of graphene is an important parameter which hampers material removal at nanometric scales. For over-threshold beam diameter below 1 µm, the intensity distribution, ie beam shape, is an important factor for the morphology of the crater.

## Conclusion

We have identified a decrease of the single-shot ablation probability of CVD graphene when the over-threshold beam diameters decreases below 1 µm, with two different behaviours depending on the presence or absence of graphene wrinkles in the vicinity of the ultrafast laser illuminated impact. Ablation probability in flat graphene islands has a step-like decrease to zero when the over-threshold beam diameter decreases below 1 µm. However, in the case of laser illumination in the vicinity of wrinkles, the ablation probability decreases gradually with decreasing input pulse energy. These results can be qualitatively understood because of the high electronic and thermal conductivities of graphene. The limitations in terms of diameter make preferable to use multiple shot illumination approaches to overcome the effect of diffusion and folding.

We also report a high dependence on the ablation behaviour and hole morphology with the laser fluence distribution. For low fluence gradients we observed that minimal hole diameter is ~1 µm for single shot even if the over-threshold is down to ~200 nm. We note that accurate control of laser fluence gradients could lead to a selective formation of folded graphene petals for potential applications requiring a precise combination of mono- and bilayer graphene.

## Methods

The samples were CVD graphene monolayer on Corning Eagle-XG AMLCD glass substrates from Graphene supermarket. The graphene monolayers were transferred on the substrate after growth.

The sample position was controlled with sub-micron precision with a 5 axis motorized translation stages and planarity was ensured to be better than 1 µm over the whole sample. The accuracy of the sample positioning is enabled by an independent imaging system including a high numerical aperture objective (NA 0.8), ensuring better than 1 µm longitudinal sectioning.

## References

[1] Bonaccorso F, Sun Z, Hasan T, Ferrari AC (2010). Graphene photonics and optoelectronics. Nature Photon..

[2] Wang W (2015). Plasmonic eigenmodes in individual and bow-tie graphene nanotriangles. Sci. Rep..

[3] Nikitin AY, Guinea F, Martin-Moreno L (2012). Resonant plasmonic effects in periodic graphene antidot arrays. Appl. Phys. Lett..

[4] Hemanouche A (2014). FIB patterning of dielectric, metallized and graphene membranes: A comparative study. Microelectron. Eng..

[5] Ponomarenko LA (2008). Chaotic Dirac billiard in graphene quantum dots. Science.

[6] Kumar P, Subrahmayan KS, Rao CNR (2011). Graphene patterning and lithography employing laser/electron-beam reduced graphene oxide and hydrogenated graphene. Mater. Express.

[7] Wang C, Morton KJ, Fu Z, Li WD, Chou SY (2011). Printing of sub-20 nm wide graphene ribbon arrays using nanoimprinted graphite stamps and electrostatic force assisted bonding. Nanotechnology.

[8] Qi ZJ (2013). Direct electron beam patterning of sub-5 nm monolayer graphene interconnects. Proc. of SPIE.

[9] Currie M (2011). Quantifying pulsed laser induced damage to graphene. Appl. Phys. Lett..

[10] Sahin R, Simsek E, Akturk S (2014). Nanoscale patterning of graphene through femtosecond laser ablation. Appl. Phys. Lett..

[11] Wetzel B, Xie C, Lacourt P-A, Dudley JM, Courvoisier F (2013). Femtosecond laser fabrication of micro and nano-disks in single layer graphene using vortex Bessel beams. Appl. Phys. Lett..

[12] Joglekar AP, Liu H, Meyhöfer E, Mourou G, Hunt AJ (2004). Optics at critical intensity: Applications to nanomorphing. Proc. Natl. Acad. Sci; USA.

[13] Mercadier L (2013). Femtosecond laser desorption of ultrathin polymer films from a dielectric surface. Appl.Phys.Lett..

[14] Yoo JH, In JB, Park JB, Jeon H, Grigoropoulos CP (2012). Graphene folds by femtosecond laser ablation. Appl. Phys. Lett..

[15] Roberts A (2011). Response of graphene to femtosecond high-intensity laser irradiation. Appl. Phys. Lett..

[16] Kalita G, Qi L, Namba Y, Wakita K, Umeno M (2011). Femtosecond laser induced micropatterning of graphene film. Mat. Lett..

[17] Stöhr RJ, Kolesov R, Xia K, Wrachtrup J (2011). All-optical high-resolution nanopatterning and 3D suspending of graphene. ACS Nano.

[18] Gil-Villalba A (2015). Deviation from threshold model in ultrafast laser ablation of graphene at sub-micron scale. Appl. Phys. Lett..

[19] Zhu W (2012). Structure and Electronic Transport in Graphene Wrinkles. Nano Letters.

[20] Ahmad M, Han SA, Tien DH, Jung J, Seo Y (2011). Local conductance measurement of graphene layer using conductive atomic force microscopy. J. Appl. Phys..

[21] Courvoisier F (2009). Surface nanoprocessing with nondiffracting femtosecond Bessel beams. Optics Lett..

[22] Bhuyan MK (2010). High aspect ratio nanochannel machining using single shot femtosecond Bessel beams. Appl. Phys. Lett..

[23] Duocastella M, Arnold CB (2012). Bessel and annular beams for materials processing. Laser Photon. Rev..

[24] Froehly L, Jacquot M, Lacourt P-A, Dudley JM, Courvoisier F (2014). Spatiotemporal structure of femtosecond Bessel beams from spatial light modulators. J. Opt. Soc. Am. A.

